# Milky pericardial effusion causing tamponade in a neonate after extracorporeal membrane oxygenation cannulation

**DOI:** 10.1093/jscr/rjad233

**Published:** 2023-05-10

**Authors:** Rosanne Thornhill, Randall Fortuna, Kayla Canteras, Steven L Raymond, Faraz A Khan, Andrei Radulescu

**Affiliations:** Department of Surgery, Loma Linda University Children's Hospital, Loma Linda, CA, USA; Division of Cardiothoracic Surgery, Loma Linda University Children's Hospital, Loma Linda, CA, USA; Loma Linda University School of Medicine, Loma Linda, CA, USA; Division of Pediatric Surgery, Loma Linda University Children's Hospital, Loma Linda, CA, USA; Division of Pediatric Surgery, Loma Linda University Children's Hospital, Loma Linda, CA, USA; Division of Pediatric Surgery, Loma Linda University Children's Hospital, Loma Linda, CA, USA

**Keywords:** neonate, tamponade, ECMO, pericardial effusion, milky

## Abstract

Cardiac tamponade is a known life-threatening complication of extracorporeal membrane oxygenation (ECMO), often secondary to hemopericardium from major vascular or cardiac perforation. We present the unique case of a neonate with a milky pericardial effusion causing tamponade after ECMO cannulation, managed successfully with pericardial window. Understanding ECMO physiology and its effect on the classic presentation of tamponade is critical and can prevent delays in diagnosis. While hemopericardium is most commonly seen in these cases, findings of a non-bloody, milky effusion should prompt further workup for infection, chylopericardium or total parenteral nutrition-associated pericardial effusion, as the appropriate management can mitigate immediate and potential long-term sequelae.

## INTRODUCTION

Extracorporeal membrane oxygenation (ECMO) remains a well-established supportive therapy for neonates with severe cardiac and respiratory failure refractory to maximal conventional management. Though lifesaving in many cases, serious potential complications remain. One rare complication, associated with significant morbidity, is inadvertent vascular or cardiac perforation during cannulation. This can result in hemopericardium and subsequent cardiac tamponade, requiring emergent intervention [1]. We present the unique case of a neonate developing cardiac tamponade within 24 h of ECMO cannulation, secondary to an unexpected milky pericardial effusion.

## CASE REPORT

This case describes a female born via cesarean section at 41 weeks gestation to a healthy 33-year-old female, Gravida 1, Para 1. Upon delivery, thick meconium was seen in utero with worsening respiratory distress of the newborn, concerning for meconium aspiration. The patient eventually required intubation and echocardiogram was performed, showing a large patent ductus arteriosus, right ventricular dysfunction and severe pulmonary hypertension, with no pericardial effusion noted. She was placed on inhaled nitric oxide and total parenteral nutrition (TPN) was initiated via umbilical central venous catheter (CVC). Because of worsening oxygenation despite optimal ventilator settings, she was transferred to our Level-3 neonatal intensive care unit for ECMO evaluation.

**Figure 1:**
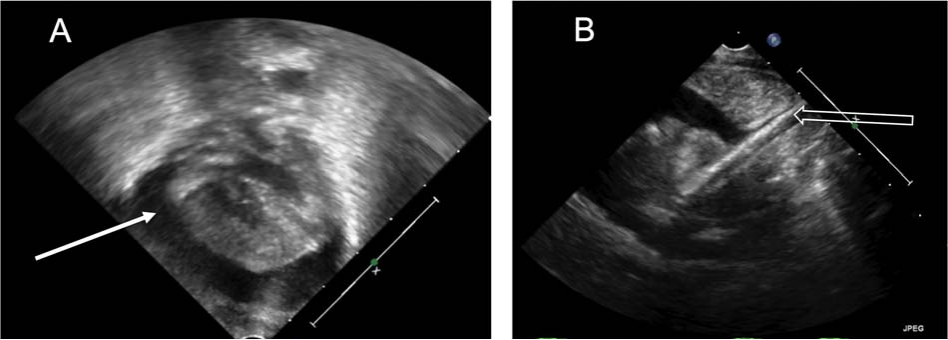
Echocardiogram. (**A**) Apical view, pericardial effusion (white solid arrow). (**B**) Subcostal view, ECMO catheter tip seen entering the right atrium (white hollow arrow).

At our institution, the patient underwent venoarterial (VA) ECMO cannulation on day of life 1, with a 12 French (Fr) venous cannula in the internal jugular vein and an 8 Fr arterial cannula in the carotid artery. Over the next 24 h, she became intermittently hypotensive, to a mean arterial pressure of 20–30 mmHg, and tachycardic, above 200 beats per minute, with decreasing ECMO flows. Despite resuscitation with blood products, she remained hemodynamically unstable with persistently low ECMO flows. Cardiac tamponade was suspected, and echocardiogram confirmed a large pericardial effusion with severely diminished biventricular systolic function (Fig. 1). With concern for possible cardiovascular perforation during ECMO cannulation, she was taken emergently for mediastinal exploration. A pericardial window was performed with drainage of 20 ml of opaque, milky fluid and immediate improvement in ECMO flows. No signs of cardiac injury, vascular perforation or hemorrhage were observed. Fluid analysis was sent (Table 1) and a right pleuro-pericardial window was created, with a 15 Fr drain placed.

**Table 1:** Patient’s pericardial fluid analysis.

**Fluid characteristics**	**Patient sample**
Color	White
Triglycerides (mg/dL)	543
RBCs (%)	5
Segmented neutrophils (%)	37
Lymphocytes (%)	1
Gram stain	No organisms
Culture	Negative
Cytology	No malignant cells

The patient continued to improve postoperatively. She was decannulated on ECMO day 5, the pericardial drain was removed the next day and she was extubated on day of life 10. Repeat echocardiogram showed normal biventricular systolic function with complete resolution of pulmonary hypertension and no recurrent pericardial effusion. She transitioned from TPN to enteral feeds on day of life 12 and was discharged home without supplemental oxygen on day of life 40.

## DISCUSSION

Recognizing cardiac tamponade in ECMO patients is challenging, as the characteristic signs are less specific, less pronounced or completely absent during ECMO [2]. The classically noted muffled heart sounds and diminished pulses are also seen with VA ECMO, as the heart is relatively empty with diminished pulsatility. Pulsus paradoxus is hardly seen as continuous flow from the ECMO pump obscures typical respiratory variations in systolic flow. Alternatively, suspicion for cardiac tamponade on ECMO should be raised with persistently low circuit flows, as the right atrial wall can compress against the venous cannula, obstructing venous return to the circuit. Supporting signs include a widened mediastinum on chest X-ray, loss of arterial wave form pulsatility, elevated central venous pressure and ongoing volume requirements to maintain circuit flows.

Echocardiogram, the diagnostic tool of choice, can be obtained quickly at bedside, with confirmed tamponade requiring urgent pericardial drainage. This rapidly improves cardiac filling, restoring venous return to the ECMO circuit and delivery of oxygenated blood systemically. While pericardiocentesis is effective, surgical drainage with pericardial window is warranted in suspected cardiovascular injury, with extension to sternotomy if hemopericardium or obvious injury is confirmed. Most cases of ECMO cannulation complicated by tamponade involve hemopericardium with a few serous effusions reported [2, 3]. To our knowledge, this is the first case reported of a milky pericardial effusion causing tamponade in a neonate following ECMO cannulation.

Neonatal milky pericardial effusions are rare, typically secondary to infection, chylopericardium or TPN via CVCs. Primary (idiopathic) chylopericardium is rare, with very few documented pediatric cases (Table 2). Patients are usually asymptomatic, with incidental cardiomegaly on chest X-ray, but can present with cough, fatigue or progressive dyspnea [4]. Treatment prioritizes reducing recurrence, decreasing the metabolic complications from persistent chyle leak and preventing mechanical sequelae, like tamponade or constrictive pericarditis. Stable patients are managed conservatively by pericardiocentesis, with strict TPN, octreotide and low-fat diets high in medium-chain triglycerides as useful adjuncts [5–7]. Surgical management is reserved for medically refractory cases or hemodynamic instability, most effective when pericardial window and thoracic duct ligation are performed [8].

**Table 2:** Cases of primary chylopericardium in the pediatric population between the years 2000 and 2022.

**Author**	**Year**	**Age**	**Sex**	**Signs/symptoms**	**Conservative management**	**Surgical management**
López-Castilla [5]	2000	2 m	M	Dyspnea, anorexia	Pericardiocentesis + D Low fat, MCT diet TPN	None
Tan [6]	2001	5 y	M	Cough, dyspnea	Pericardiocentesis + D Low fat, MCT diet	VATS + PW + L
Ossiani [11]	2003	6 wk	F	Fussiness, crying, perioral cyanosis	Pericardiocentesis + D Low fat, MCT diet	Left thoracotomy + PW + L
Ossiani [11]	2003	13 y	M	Cough	Pericardiocentesis + D Low fat, MCT diet	Left thoracotomy + PW + L
Hattori [12]	2011	15 y	F	Asymptomatic	Pericardiocentesis + D	VATS + PW + L
Rivera-Beltrán [13]	2013	16 y	M	*Initially: asymptomatic* 1 year later: chest pain, dizziness	*Lasix, Prednisone* Low fat, MCT diet Octreotide	1.P, D 2.Open abdominal PW + L
Karakurt [7]	2014	4 y	M	Dyspnea, tachypnea, cardiac tamponade	Pericardiocentesis + D TPN Octreotide	1.VATS + PW + D 2. L
Courtney [14]	2014	10 y	M	Right upper quadrant pain, headache, dizziness ^a^Dyspnea, fatigue, chest pain	Pericardiocentesis	PW + L (incomplete) ^b^Thoracic duct embolization
Jasani [15]	2018	2 y	U	Dyspnea	Pericardiocentesis Low fat, MCT diet	VATS + P
Jasani [15]	2018	4 y	U	Dyspnea	Pericardiocentesis Low fat, MCT diet	1. VATS + P 2. Open abdominal PW + D + L

Abbreviations: wk, weeks old; m, months old; y, years old; M, male; F, female; U, unspecified sex; MCT, medium-chain triglycerides; VATS, video-assisted thoracoscopic surgery; P, pericardiectomy; PW, pericardial window; D, pericardial drain; L, ligation of thoracic duct. ^*^Symptoms; ^*^^*^management of recurrence.

Alternatively, many milky effusions initially suspicious for chylopericardium were found to be CVC-infused TPN in the pericardial space [9]. CVC-related pericardial effusion with tamponade occurs in 1–3% of neonates, with a 30–50% mortality [1]. Originally thought to be secondary to right atrial perforation by a deep CVC, persistent events despite appropriate catheter position leave the true underlying mechanism unknown. Nevertheless, early diagnosis is critical, requiring prompt pericardial drainage, TPN discontinuation and CVC removal [1, 10].

The etiology of our patient’s milky pericardial effusion remains a mystery. Table 3 compares our fluid sample to other characteristically milky pericardial effusions. Negative cultures with no organisms on gram stain rule out infection. Despite high triglycerides, the lack of lymphocyte predominance makes chylopericardium less likely. Although a TPN-associated infusion is possible, lack of recurrence, despite continuing TPN with the same CVC, seems unlikely. Though she responded well to primary surgical drainage without recurrence, this case highlights the importance of a comprehensive differential, as second steps, further diagnostics and adjunctive treatments vary significantly.

**Table 3:** Comparison of milky pericardial fluid characteristics by etiology [4, 9].

**Fluid characteristics**	**Patient sample**	**Infection**	**Chylopericardium**	**TPN-associated effusion**
Gross appearance	White	White/yellow (Turbid)	White/milky	White/milky
Triglycerides	High	Low	High	High
Segmented neutrophils	Mod	High	Low	Low
Lymphocytes	Low	Low	High	Low
Gram stain	No organisms	Positive	Negative	Negative
Culture	Negative	Positive	Negative	Negative

Cardiac tamponade can be life-threatening and is challenging to diagnose in patients on ECMO. A high index of suspicion, familiarity with ECMO physiology and prompt echocardiography prevents diagnostic delays, decreasing morbidity and mortality for neonates. Comprehensive workup for milky pericardial effusions can guide further steps in management, reducing immediate and potential long-term complications.

## CONFLICT OF INTEREST STATEMENT

None declared.

## FUNDING

None.

## DATA AVAILABILITY

The data supporting the conclusions of this article are included in the article.
